# Relationship Between Mutans Streptococci and Lactobacilli in the Oral Cavity and Intestine of Obese and Eutrophic Children With Early Childhood Caries—Preliminary Findings of a Cross-Sectional Study

**DOI:** 10.3389/fped.2020.588965

**Published:** 2020-12-11

**Authors:** Claudia Maria dos Santos Pereira Indiani, Karina Ferreira Rizzardi, Camila Lopes Crescente, Carolina Steiner-Oliveira, Marinês Nobre-dos-Santos, Thaís Manzano Parisotto

**Affiliations:** ^1^Laboratory of Clinical and Molecular Microbiology, University São Francisco - USF, Bragança Paulista, Brazil; ^2^Department of Pediatric Dentistry, Piracicaba Dental School, State University of Campinas - UNICAMP, Piracicaba, Brazil

**Keywords:** obesity, bacteria, children, preschool, early childhood caries

## Abstract

This brief communication assessed whether there was any relationship between the counts of lactobacilli (LB) and mutans streptococci (MS) in the oral cavity and intestine of obese and eutrophic children with early childhood caries (ECC). Seventy-eight preschoolers were assigned into the following groups: 1. obese children with ECC (OECC), 2. eutrophic children with ECC (EECC), 3. obese caries-free children (OCF), and 4. eutrophic caries-free children (ECF). The diagnosis of obesity and ECC was based on the World Health Organization criteria. Dental plaque and fecal samples were collected to assess the counts of MS and LB using selective media. Data were evaluated by Poisson regression analysis, Wilcoxon test, and Sign test. Microbial indicators of ECC in obese children were MS counts in the intestine [rate ratio (RR): 4.38] and presence of LB in the oral cavity (RR: 2.12). The indicators in eutrophic children were MS levels and the presence of LB, both in the oral cavity (RR: 6.35/1.50) and intestine (RR: 2.35/2.38) (*p* < 0.05). The comparison between MS levels in the mouth and in the intestine revealed significant differences only in the ECF group (*p* = 0.04). Regarding LB presence in the mouth vs. in the intestine, except for the OCF group (*p* = 0.03), no other statistical differences were found. Our preliminary findings highlighted that the levels of MS and the presence of LB in the oral cavity, as well as in the lower gastrointestinal tract were associated with ECC. Moreover, obesity was found to influence this relationship.

## Introduction

Early childhood caries (ECC) remains highly prevalent in children, especially among those in deprived communities. The influence of this chronic multifactorial disease on children up to 71 months of age is significant in the society (1, 2).

While the key factors involved in the etiology of ECC are well known, there are specific risk indicators that should be explored. In the microbial pathogenesis of ECC, the role played by mutans streptococci (MS) and lactobacilli (LB) should be highlighted (3–10). These acidogenic and aciduric bacteria can ferment dietary substrates and produce organic acids, which lead to dental tissue demineralization (1, 11). MS and LB belong to the phylum Firmicutes, which is one of the most prevalent phyla in the oral cavity (8, 12). Interestingly, an increase in the number of bacteria belonging to this phylum has been observed in childhood obesity (13–15), a noncommunicable disease associated with microbial dysbiosis (16–18) in the same manner as caries (19, 20). In addition, a recent systematic review and meta-analysis indicated that obese children are more vulnerable to ECC (21).

The mouth is a significant gateway to the human body, and in the first stage of the digestive process, foods are broken down by the teeth and mixed with saliva to form a bolus, which then moves into the gastrointestinal tract. Remarkably, bacteria colonizing the oral cavity have a substantial chance of spreading to contiguous epithelial surfaces to neighboring sites along the food pathway (22, 23). Thus, it may be hypothesized that some groups of oral bacteria could also be found in the intestine, and to the best of our knowledge, there is no scientific evidence available on this subject in early childhood (24).

This preliminary study aimed to assess the possible relationship between counts of LB and MS in the oral cavity and in the lower gastrointestinal tract of obese and eutrophic children with ECC.

## Materials and Methods

### Experimental Design

The sample size was estimated based on the results of a pilot study involving five obese preschoolers with ECC (main conditions to be studied) that showed a 25.1 × 10^4^ ± 30.0 × 10^4^ colony-forming units per milliliter difference in the MS levels (main variable to be studied) when the mouth and the intestine were compared. These children were not included in the study, and these data were not published. A minimum of 18 children per group was required considering a 5% significance level, 0.78 effect size (25), and 80% power. Due to possible dropouts, 88 preschoolers of both sexes were enrolled and assigned to the following groups: 1. obese children with ECC (OECC, *n* = 22); 2. eutrophic children with ECC (EECC, *n* = 22); 3. obese caries-free children (OCF, *n* = 22); and 4. eutrophic caries-free children (ECF, *n* = 22).

Healthy children (3–5 years old) attending public preschools in the city of Bragança Paulista-SP, Brazil, with access to fluoridated tap water (0.7 ppm), those who used fluoridated dentifrice, and whose guardians signed the informed written consent form, according to The Code of Ethics of the World Medical Association (Declaration of Helsinki) (São Francisco University Ethics Committee approval: 42997115.4.0000.5514) were included in this study. The exclusion criteria were as follows: presence of syndromes/systemic diseases, children with enamel defects, and those who were taking antibiotics during or up to 30 days before the sample collection period. All enrolled children were from similar socioeconomic backgrounds and spent the entire day in the preschool, where the meals provided were standardized.

### Early Childhood Caries Diagnosis

The clinical examinations for diagnosis of ECC were performed by two calibrated dentists. First, theoretical discussions using clinical images were conducted to provide instructions on the diagnostic criteria to the examiners. Subsequently, for the training, 10 children were examined by a “gold standard” and second by the examiners. Third, the dentists evaluated another group of 10 children, and the Kappa coefficient was calculated, indicating an excellent agreement of 0.86.

The children were seated by a window and were examined after each tooth was cleaned and dried with sterile gauze. Disposable gloves and masks, a focusable flashlight, a mouth mirror, and a ball-ended dental probe were used to diagnose dental caries. The diagnosis was made according to the World Health Organization criteria (26), with the inclusion of the active white spot lesions (4). A chalky, rough, white spot lesion without a surface breakdown, close to the soft tissue margin where the biofilm accumulates, or extending along the walls of the fissure on the occlusal surface, was considered as an active white spot lesion. The units of evaluation used in the clinical exams were the number of decayed (cavitations + active white spot lesions), missing, or filled surfaces (dmfs) of the primary teeth.

### Nutritional Status Assessment

The anthropometric assessment included the measurement of body weight and height. A portable and calibrated digital scale (100 g of precision) was placed on a flat surface, and children were weighed while standing still at the center of the scale with the Camper Plane parallel to the floor and their feet slightly apart. The children were placed barefoot after removing heavy clothes. Although the children were weighed while wearing only light uniforms, 100 g was subtracted from the total body mass to avoid bias. Height was measured using a tape affixed on a wooden board at a 90° angle with the ground. The children were placed erect with the Camper Plane parallel to the floor. The weight and height of the children were used to calculate the body mass index [BMI = weight (kg)/height^2^ (m)]. The BMI, age in months, and sex were used to classify the children as eutrophic or obese, based on the World Health Organization criteria. Children with Z-scores > +3, or ≥−2 but ≤+1 were classified as obese and eutrophic, respectively (27).

### Dental Biofilm and Fecal Samples Collection and Analysis

Supragingival dental biofilm was collected from the buccal surfaces of the upper primary incisors, except from the interior of the cavities, at least 1 h before or after food intake. A sterilized plastic handle (Greiner, Frickenhausen, Germany) was used to standardize the collection procedures and the biofilm quantity. The collection stopped when the handle (circular opening of 1-μl capacity) was full (4). The dental biofilm was inserted into microcentrifuge tubes containing 0.9% sterile saline solution, which were stored in ice until analyses.

Disposable plastic containers were used for collection of fecal samples. The guardians were instructed to collect the samples of the children at their homes (without touching the inside of the toilet) and bring them to the preschool within a 12-h period. Samples were kept refrigerated at all times (in hermetically sealed plastic bags placed inside refrigerators at home or into refrigerated boxes at school) until the microbiological analyses at São Francisco University. For this analysis, the same sterilized plastic handle of the biofilm sample collection was used to standardize the fecal quantity to be inserted into microcentrifuge tubes containing 0.9% sterile saline solution.

Thus, for the microbiological analyses, equal amounts of biofilm and feces (≅5 mg) were serially diluted (10^−1^-10^−5^) and cultured in triplicates into the following media: Mitis salivarius agar (Difco, Sparks, MD) containing 0.2 U/ml of bacitracin (Sigma, Poole, UK) and 20% sucrose (for MS), Rogosa agar (Difco, Sparks, MD) supplemented with 0.13% glacial acetic acid (for LB), and brain heart infusion agar (Difco, Sparks, MD) containing 5% defibrinated sheep blood (for total microorganisms). Plates were incubated for 48 h at 37°C in candle extinguishing jars. After this period, all plates were photographed, and the number of colony-forming units (CFU) was counted using a computer program (CFU count, Jaú, SP, Brazil). The results were expressed as CFU/ml.

### Statistical Analysis

The analyses were conducted considering a 5% significance level, and the statistical programs used were SPSS 16.0 (SPSS Inc., Chicago, IL, USA) and JASP 0.9.1 (University of Amsterdam). A model comprising the studied microbial indicators of ECC in obese and eutrophic preschoolers was constructed after Poisson regression analysis, in which the main outcome was dmfs. In this analysis, rate ratio was considered as a measure of effect size (1.22 = small; 1.86 = medium; 3.00 = large) (28). MS levels were adjusted by dividing them by the total microorganism levels (5, 7). LB was assessed based on the presence or absence of the bacteria due to their low CFU/ml counts (4). As the Shapiro–Wilk test revealed that the data did not follow a normal distribution, counts of MS in the oral cavity and intestine within the groups were compared using the Wilcoxon test, and the Sign test was used to compare the presence of LB in the oral cavity and intestine within the same group. While rank-biserial correlation was considered a measure of effect in the Wilcoxon test (<0.1 = trivial; 0.1 = small; 0.3 = medium; 0.5 = large) (29–31), odds ratio was considered for the Sign test (1.32 = small; 2.38 = medium; 4.70 = large) (28).

## Results

Of the 88 children assessed initially, 10 were excluded [four children in each of the obesity groups (OECC and OCF) and two in the ECF group] because their fecal samples were not brought in the required period (n = 4) or were not in accordance with the instructions (*n* = 6). Thus, the final sample comprised 78 children assigned into the following groups: OECC (*n* = 18), EECC (*n* = 22), OCF (*n* = 18), and ECF (*n* = 20).

Table 1 shows the microbial indicators of ECC in eutrophic and obese preschoolers. Considering obese children, the significant indicators were MS counts in the intestine [rate ratio (RR): 4.38] and presence of LB in the oral cavity (RR: 2.12). Furthermore, the statistically significant indicators of ECC in eutrophic children were MS levels and the presence of LB, in the oral cavity (RR: 6.35/1.50) and in the intestine (RR: 2.35/2.38).

**Table 1:** Microbial indicators of early childhood caries (ECC) in eutrophic and in obese preschoolers.

		**Model 1—Indicators of ECC in obese preschoolers**		**Model 2—Indicators of ECC in eutrophic preschoolers**	
	**Parameters**	**Rate ratio (95% CI)**	***p*-value**	**Rate ratio (95% CI)**	***p*-value**
Mutans streptococci levels^#^ (CFU/ml)	Oral cavity	1.69 (0.02–100.00)	0.82	6.35 (1.54–26.20)	0.01*
	Gut	4.38 (1.98–9.68)	<0.01*	2.35 (1.31–4.19)	<0.01*
Lactobacilli (presence^reference^/absence)	Oral cavity	2.12 (1.16–3.85)	0.01*	1.50 (1.02–2.32)	0.04*
	Gut	1.36 (−0.79–2.33)	0.27	2.38 (1.54–3.70)	<0.01*

Poisson regression model; main outcome: early childhood caries (number of decayed, missing, or filled surfaces of the teeth). *Statistically significant at p < 0.05. CI, confidence interval. Model 1 (n = 36): Omnibus test: likelihood ratio chi-square = 15.99, freedom degree 4, significance 0.003; Model 2 (n = 42): Omnibus test: likelihood ratio chi-square = 40.82, freedom degree 4, significance 0.000.

# *MS levels were adjusted by dividing them by the total microorganism levels. Rate ratio was considered as a measure of effect size: 1.22 (small), 1.86 (medium), 3.00 (large) (28)*.

The comparison between MS levels in the mouth and in the intestine, with respect to all groups studied, revealed significant differences only in the ECF group (*p* = 0.04—Table 2) with higher amounts in the gut. In addition, the effect size of this difference was large (rank-biserial correlation = 0.55). Moreover, regarding LB presence in the mouth vs. in the intestine, its presence was significantly more frequent in the gut only in the OCF group (*p* = 0.03—effect size 1.80—Table 3). Considering the other groups, no statistical differences in LB frequency were found between the investigated niches (*p* > 0.05).

**Table 2:** Comparison of mutans streptococci levels in the oral cavity and in the lower gastrointestinal tract of preschoolers, with respect to early childhood caries (ECC) and obesity.

	**Mutans streptococci levels**^*******#*******^ **(CFU/ml)**			
	**Oral cavity** ** Median (interquartile** ** deviation)**	**Gut** ** Median (interquartile** ** deviation)**	***p*-value**	**Effect size**
Eutrophic caries-free children (*n* = 20)	0.01 (0.07)	0.07 (0.15)	0.04*	0.55
Obese caries-free children (*n* = 18)	0.05 (0.07)	0.01 (0.03)	0.22	−0.34
Eutrophic children with ECC (*n* = 22)	0.07 (0.08)	0.05 (0.16)	0.86	0.09
Obese children with ECC (*n* = 18)	0.05 (0.08)	0.02 (0.07)	0.44	−0.22

# MS levels were adjusted by dividing them by the total microorganism levels. *Statistically significant difference between the oral cavity and gut inside each group—Wilcoxon test (α = 0.05), comparison in line. Rank-biserial correlation was considered as a measure of effect size: <0.1 (trivial), 0.1 (small), 0.3 (medium), 0.5(large) (29–31).

**Table 3:** Presence or absence of lactobacilli in the oral cavity compared with that in the lower gastrointestinal tract of preschoolers, with respect to early childhood caries (ECC) and obesity.

			**Dental plaque**	**Feces**		
			**Number of children**		***p*-value**	**Effect size**
Eutrophic caries-free children (*n* = 20)	Lactobacilli	Absent	16	11	0.06	0.20
		Present	4	9		
Obese caries-free children (*n* = 18)		Absent	14	7	0.03^*^	1.80
		Present	4	11		
Eutrophic children with ECC (*n* = 22)		Absent	9	8	0.50	0.57
		Present	13	14		
Obese children with ECC (*n* = 18)		Absent	12	11	0.50	1.43
		Present	6	7		

* *Statistical difference between the oral cavity and gut inside each group—Sign test (α = 0.05). Odds ratio was considered as an effect size: 1.32 (small), 2.38 (medium), 4.70 (large) (28)*.

## Discussion

To the best of our knowledge, this study is the first to provide preliminary scientific evidence based on the counts of MS and the presence of LB in the oral cavity and in the lower gastrointestinal tract of children affected by ECC and obesity. In obese children, it was observed that every CFU/ml increase in MS in the intestine was associated with a 4.38 times increase in the number of ECC lesions. Similarly, among eutrophic preschoolers, 6.35 and 2.35 times increases in the number of carious lesions were observed for every CFU/ml increase in MS in the oral cavity and the gut, respectively (Table 1). In addition, considering the two investigated niches, the MS levels were similar in all groups (*p* > 0.05), with the exception of children in the ECF group who showed significantly higher MS counts in the intestine than in the mouth (Table 2). MS are intimately related to the initiation and/or progression of carious lesions (4–7, 9, 32–36). The finding that lower gastrointestinal tract levels of MS were associated with caries is based on the fact that bacteria colonizing the mouth can spread to contiguous epithelial surfaces to neighboring sites along the pathway of food (22, 23). Obesity and ECC have been linked to microbial dysbiosis (16, 19, 20). In the gut of obese children, bacteria from the Firmicutes phylum play a role in amplifying this dysbiosis (13, 14, 18). Intriguingly, MS, which is the main pathogen of ECC (1, 9, 11), belongs to the Firmicutes phylum.

Remarkably, the gut microbiome development may begin *in utero*. During pregnancy, the transfer of bacteria may occur from mother to baby (via blood stream, placenta, and amniotic fluid) (37) and can be seen in the meconium (38). Changes in the microbiota of meconium and the newborn stool samples could be influenced even by maternal gestational diet (39, 40). For instance, increased fruit intake was connected to a high *Streptococcus/Clostridium* bacterial group among infants vaginally born. After the prenatal period, the mode of delivery is of prime importance for the initial colonization of the gut. While vaginally delivered newborns harbored increased abundance of *Streptococcus*, cesarean section-born infants show low *Streptococcus* genera (40). Birth gestational age also influenced the gut microbiome, as preterm infants are submitted to antibiotics, hospital stay, and enteral feeding, in addition to their immaturity (41). Subsequently, with the introduction of breast milk/formula (oral feedings), the gut bacteria have additional stimulus, and later, with weaning to solid foods, a more adult-like microbiota starts to develop, being further dominated by Firmicutes and Bacteroidetes (38, 42).

The recent investigation by Kennedy et al. (43) involving a cohort of 59 children sampled at 6, 12, and 24 months highlighted that birth characteristics, breastfeeding, antibiotic use, and salivary cortisol play a significant role in the development of the oral microbiota in early childhood. In addition, they revealed a dramatic shift in the oral microbiome composition from 6 to 24 months, coinciding with the introduction of solid foods and with the period of primary tooth eruption, which provide new micro-environments for colonization. Moreover, the similarity with the maternal microbiota increases over time, leading to a more adult-like composition at 24 months (43). Differences in operational taxonomic units of bacterial genera belonging to the Firmicutes phylum were already described between a group of children fed with breast milk and a group fed with formula, combined or not with breast milk (44). In addition, when the oral microbiome data were compared with ECC development, *Streptococcus mutans* was identified as the most discriminatory taxa associated with disease (45), and their levels could be a surrogate marker for frequent sugar intake by children. High sugar exposure leads to dysbiosis of the dental biofilm microbiota, resulting in acidogenic and aciduric bacteria (20, 45).

The Poisson regression analysis indicated that the presence of LB in the oral cavity [rate ratio (RR): 2.12] was associated with ECC in obese children. Furthermore, the presence of LB in the oral cavity (RR: 1.50) as well as in the intestine (RR: 2.38) was closely associated with carious lesions in eutrophic preschoolers (Table 1). In addition, the oral condition was not significantly different from that of the gut in obese or eutrophic preschoolers with ECC (*p* > 0.05) (Table 3). These results may be explained by the fact that caries, particularly cavitated lesions, act as ecological niches for LB colonization. Our findings are in line with previous studies that reported associations between caries and the LB presence (1, 4, 5, 19, 34, 46). A clinical review by Caufield et al. (46) suggested the oral cavity as the main pool of LB for the gastrointestinal tract. Interestingly, LB can resist the digestive process and remain viable in the gut (47), where they are frequently identified (48). The exact mechanism by which these bacteria remain alive in the gastrointestinal tract has not yet been elucidated. However, LB can survive at extremely low pH, unlike other less acid-tolerant microorganisms. Many strains of LB are present in dairy products and are able to change the microbiota of the gut. A systematic review and meta-analysis reported that LB was significantly associated with weight modification in humans, depending on the strain (49). A study by Nobili et al. (50), involving older children (average age of 11.45 years), showed increased counts of *Lactobacillus* spp. in fecal samples of obese children compared to children in the control group. Specific conditions such as formula feeding and cesarean section might negatively influence the development of the normal microbiota, favoring a decrease in the levels of LB and Bifidobacterium and an increase in the levels of Clostridia (51).

The current study has some limitations. All enrolled children belonged to similar socioeconomic backgrounds and spent most of the day at the public preschools, where the meals provided were the same for all children. Significant differences in the socioeconomic status and diet between the groups, choice of food during pregnancy, gestational age, mode of delivery, lactation method, antibiotics usage, and genetics would provide additional information to understand the relationship between ECC, obesity, and oral/intestinal microbiota. Moreover, future larger studies involving longitudinal designs, which consider the child's response to a certain factor during the disease process, should be conducted to reinforce our findings, and our preliminary results should be used to design these studies.

## Conclusion

It may be concluded that the levels of MS and the presence of LB in the oral cavity as well as in the lower gastrointestinal tract are closely associated with ECC. Moreover, obesity was found to influence this relationship.

## Data Availability Statement

The raw data supporting the conclusions of this article will be made available by the authors, without undue reservation.

## Ethics Statement

The studies involving human participants were reviewed and approved by São Francisco University Ethics Committee. Approval number: 42997115.4.0000.5514. Written informed consent to participate in this study was provided by the participants' legal guardian/next of kin.

## Author Contributions

CI performed the collection of the clinical data, the data interpretation, and the writing of the manuscript. CC and KR performed the collection of the clinical data and the writing of the first draft. MN-S and CS-O critically reviewed the manuscript, leading to the final version. TP was responsible for coordinating the study, for the design, the data interpretation, and also for writing the paper. All authors contributed to the article and approved the submitted version.

## Conflict of Interest

The authors declare that the research was conducted in the absence of any commercial or financial relationships that could be construed as a potential conflict of interest.
